# Embodiment of infinity in mathematics

**DOI:** 10.3389/fpsyg.2023.1321940

**Published:** 2024-01-24

**Authors:** Omid Khatin-Zadeh, Danyal Farsani, Zahra Eskandari

**Affiliations:** ^1^School of Foreign Languages, University of Electronic Science and Technology of China, Chengdu, China; ^2^Department of Teacher Education, Norwegian University of Science and Technology, Trondheim, Norway

**Keywords:** iterative embodiment, infinity, mathematical concepts, gestures, mathematics education

## Abstract

In this article, we discuss the embodiment of infinity as one of fundamental concepts in mathematics. In contrast to the embodiment of many other mathematical concepts, the embodiment of infinity is an endless dynamic process. In embodying +∞, an object moves rightward toward a previously-set limit and passes it. Then, a new limit is set on the right side of the moving object. The moving object continues its movement and passes it as well. The moving object can pass any limit. In other words, there is no impassable limit for it. In embodying -∞, a similar process happens but the movement is leftward. Embodiment of infinitely small quantities has a basic similarity to the embodiment of infinitely large quantities, although it is different in some respects. We call the embodiment of infinity as *iterative embodiment*. It is iterative because the process of setting a new limit and passing it is repeated endlessly. Finally, it is suggested that in the process of embodying infinitely large and infinitely small quantities, the visual system and the motor system play important roles, as this process involves spatial concepts and movement.

## 1 Introduction

Mathematics is seen as one of the most abstract subjects of science and a collection of intangible or disembodied ideas (e.g., Boaler et al., 2016; Alberto et al., 2022). In traditional textbooks, mathematics objects and concepts are represented in terms of abstract symbols. Since these abstract symbols are detached from sensorimotor experiences, many people find mathematics unreal, intangible, and difficult. For these people, it may be difficult to imagine how abstract mathematical symbols are associated with physical world and what objects they stand for. Representing mathematical concepts and relations in terms of abstract symbols makes mathematics more abstract and maybe more difficult to learn. However, using abstract symbols is unavoidable because these symbols have a fundamental function in mathematical cognition. Using abstract symbols allows people to express general rules and patterns that apply across a wide range of phenomena. In fact, abstract symbols do not represent physical features of concepts. Abstract symbols are used to represent general or structural relationships. Using abstract mathematical symbols is a way to abstract a wide range of superficially/physically different phenomena into a single general rule. In other words, phenomena that are superficially/physically different in appearance but are similar at a deep structural level can be described by a single general rule of abstract symbols. Since mathematical subjects are often discussed in terms of abstract symbols in mathematics textbooks, many learners are faced with difficulties to digest these generalities. Luckily, embodied mathematics teaching and learning has offered a solution to this problem (e.g., Radford, 2009; Sinclair and Heyd-Metzuyanim, 2014; Dackermann et al., 2017; Nemirovsky et al., 2020; Rosa et al., 2020; Rosa and Farsani, 2021; Farsani and Villa-Ochoa, 2022). At least, it can be said that this approach has a lot of potentials to overcome the difficulties that learners are faced with when trying to learn mathematics. Within this approach, mathematical concepts are represented in terms of embodied representations. Embodied representations of mathematical concepts can be perceived through sensorimotor systems. Therefore, when abstract mathematical concepts are represented in terms of their embodied representations, sensorimotor resources are actively employed. This allows people to ground abstract mathematical concepts into the physical environment through embodied representations and sensorimotor systems.

Embodied representations of mathematical concepts can be in a variety of forms, such as physical objects, graphs, motion events, and body movements. Some embodied mathematical representations have been widely discussed in the literature of the field. Representing mathematical functions in terms of graphs in the Cartesian coordinate system is one of such cases. Graphical representation of a mathematical function is the embodied representation of the function because there is a logical relationship between this graphical representation and the concept it refers to. In fact, graphical representation offers a visually-perceivable representation of the idea that is expressed by a mathematical function. In this sense, graphical representation of a function (in the Cartesian coordinate system) is different from formal representation that is expressed as *y* = *f(x)*. Formal representation of a function has a primarily arbitrary relationship with the concept it refers to. Since the graphical representation of the function is visual, the visual system is actively employed to ground this concept in the visually-perceivable environment (Khatin-Zadeh, 2022). Even in the absence of graphical representation of the function when people think about the function in terms of its graphical representation, the visual system can actively be employed as a supporting tool to process the function. This is supported by the findings of a study conducted by Farah (1989) that suggest mental imagery and real visual perception involve the activation of the same areas in the visual system in prestriate occipital cortex, parietal, and temporal cortex. Findings along the same line have been reported by some other studies (e.g., Bartolomeo, 2008; Hamamé et al., 2012; Liu et al., 2022; Spagna, 2022).

Representing mathematical concepts in terms of fictive motion has also been discussed in some works (Núñez, 1998, 2008, 2009; Lakoff and Núñez, 2000; Marghetis and Núñez, 2013). Many mathematical concepts such as basic arithmetic operations, function, limit, and continuity are described in terms of fictive motion in mathematics discourse (e.g., Núñez, 1998; Lakoff and Núñez, 2000; Marghetis and Núñez, 2013), although they are inherently non-motion concepts and are defined by abstract mathematical symbols (Khatin-Zadeh et al., 2022c). Based on the embodied metaphor processing (Gallese and Lakof, 2005), understanding these mathematical concepts in terms of fictive motion involves mental simulation of fictive motion and the activation of the motor system in this mental simulation. This is supported by some empirical evidence. Results of one study showed that MT+, a brain region that responds to perceived motion, is activated during the processing of fictive motion sentences (Saygin et al., 2010). Yang and Shu (2016) found that parahippocampal gyrus, an area that is involved in spatial and motion processing, is activated during processing fictive motion sentences. Two other neuroimaging studies have also provided evidence that supports the role of the motor system in the processing of fictive motion sentences (Romero Lauro et al., 2013; Johari et al., 2021). Further evidence has been provided by studies that have shown the similarity of functional neuroanatomy associated with executed and imagined body movements (e.g., Lacourse et al., 2005; Mizuguchi and Kanosue, 2017). Since mathematical concepts that are described in terms of fictive motion can be simulated by body gestures, it has been suggested that the motor system can actively be employed to process these concepts and ground them in the concrete environment (Khatin-Zadeh et al., 2021, 2022b,c). In fact, when an abstract mathematical concept is described in terms of a fictive motion, it can be simulated by a real body gesture or an imagined body gesture. In both cases, the motor system can play a role in the processing of the concept. In addition to neuroimaging evidence, there is some behavioral evidence suggesting that processing fictive motion sentences involves simulating fictive motions (for a review, see Matlock, 2010). Even when a mathematical concept such as function is described in terms of a visual representation in the Cartesian coordinate system, it can be simulated as a fictive motion. Khatin-Zadeh et al. (2022a) suggest that visual representation of a function can be seen as the trace of a fictive motion, and this fictive motion can be mentally (and gesturally) simulated. In this way, even a static visual representation of a function can be represented and embodied as a fictive motion.

In the following section, we briefly review some works that have discussed the embodiment of numbers, arithmetic operations, limit, and continuity in terms of space, spatial concepts, and motion. These fundamental concepts have a key role in defining many mathematical concepts. Then, we discuss an iterative process through which infinitely large and infinitely small quantities are embodied. This process includes a moving object that repeatedly passes a set of dynamic boundaries set in the space.

## 2 Embodiment of mathematical concepts in terms of fictive motion

### 2.1 Embodiment of numbers

Numbers are perhaps the most basic concepts in mathematics that are described in terms of spatial concepts and fictive motion. In mathematics, numbers are represented in terms of points on an axis. This axis has an origin that represents zero. The positive and negative numbers are represented in terms of points on the right side (rightward movement) and left side (leftward movement) of the origin, respectively. For example, +3 is represented by a three-unit rightward movement from the origin, and − 4 is represented by a four-unit leftward movement from the origin. This means that numbers are embodied in terms of spatial concepts and directional fictive motions on an axis. This is also the case with the embodiment of magnitude of numbers. Smaller numbers are often associated with left side or leftward movement and larger numbers are associated with right side or rightward movement (e.g., Dehaene et al., 1990, 1993). This has been called spatial-numerical association. Spatial-numerical association has been confirmed by some experiments conducted with various cognitive tasks and stimuli (Fischer, 2018). Results of one experiment showed that after central presentation of small numbers, probes presented on the left side were detected faster (Fischer et al., 2003). In another study, after being exposed to smaller numbers, participants produced faster left-sided movements (Daar and Pratt, 2008). In both studies, larger numbers showed a stronger association with the right side. This suggests a spatial and motoric association between small numbers and left space (leftward movement), and also an association between large numbers and right space (rightward movement). All these findings suggest that rightward and leftward fictive motions play an active role in the processing of numbers. Therefore, it has been suggested that mental simulation of these fictive motions and the motor system play a role in number processing (e.g., Khatin-Zadeh et al., 2021).

### 2.2 Embodiment of arithmetic operations

In elementary mathematics, the arithmetic operations of addition and subtraction are represented and embodied in terms of rightward and leftward movements on an axis of numbers, respectively. For example, the addition (−3) + (+5) is described in terms of a five-unit fictive rightward movement that starts from the point of −3 and ends at the point of +2. The subtraction (−9) – (+7) is described in terms of a seven-unit fictive leftward movement that starts from the point of −9 and ends at the point of −16. The associations between addition and rightward movement and between subtraction and leftward movement have been supported by some empirical evidence. For example, results of one experiment showed that people tend to point to the right side after solving an addition problem and to the left side after solving subtraction problems (Pinhas and Fischer, 2008; Pinhas et al., 2014). In another study, Masson and Pesenti (2014) found that solving addition problems induced attentional shift to the right space and solving subtraction problems induced attentional shift to the left space. Such findings suggest that the arithmetic operations of addition and subtraction are embodied as fictive motion in the space. Therefore, these operations can be processed and performed by mental simulation of fictive motions and the active role of the motor system in this simulation.

### 2.3 Embodiment of limit

According to a formal definition in mathematics textbooks, limit of the mathematical function *f(x)* at *c* is defined as follows:

∀ ε > 0, ∃ δ > 0, such that if 0 < ∣x - c∣ < δ, then ∣f(x) - L∣ < ε.

Number, absolute value, and the arithmetic operation of subtraction are three basic mathematical concepts that are used to present a formal definition of limit. In this definition, these three concepts are represented by mathematical symbols. Limit of function and the three concepts that are used to present a formal definition of it are inherently non-motion and do not have any kind of relationship with motion events in the real world (Khatin-Zadeh et al., 2021). However, in informal mathematics discourse, limit of a mathematical function is described as a relationship between two movements (Marghetis and Núñez, 2013; see also Núñez, 1998). In this informal description, $\lim_{x\to c}f\left(x\right)=L$ is defined as follows:

“As the moving point *x* moves toward the fixed point *c* and the distance between *x* and *c* becomes smaller than any small distance, the moving point *f(x)* moves toward the fixed point *L* and the distance between *f(x)* and *L* can become smaller than any small distance.”

This dynamic system includes two motion events. Each motion event includes a moving point and a fixed point. The important point in this dynamic system is the relationship between these two events. That is, the distance between *f(x)* and *L* is dependent on the distance between *x* and *c*.

The definition of formal representation of limit is based on and described by non-motion concepts, while the definition of its informal representation is based on and described by motion events. The formal and informal representations of limit are inherently the same, although the elements involved in each definition are very different from the elements involved in the other. However, the formal definition, which may seem to be intangible and detached from real perceivable world, can be understood in terms of the informal definition, which is based on physical objects. Therefore, the intangible representation of limit is described and understood in terms of tangible representation that can be perceived through sensorimotor systems.

### 2.4 Embodiment of continuity

The formal definition of continuity of the function *f(x)* at *c* is based on the formal definition of the function at *c*. According to this definition, the function *f(x)* is continuous at *c* if three conditions are satisfied: (1) *f(x)* must be defined at *c*; (2) *f(x)* must have a limit at *c*; (3) this limit must be equal to *f(c)*. This is the most common and standard definition of continuity in mathematics textbooks. Like the formal definition of limit, this definition of continuity is totally based on abstract mathematical concepts that are represented by mathematical symbols. The formal definition of continuity can be represented in terms of an embodied motion-based representation. The embodied motion-based representation of continuity is much easier to understand than the formal symbol-based representation (Khatin-Zadeh and Yazdani-Fazlabadi, 2023). To create a context for presenting the embodied motion-based representation of the continuity of the function *f(x)* at *c*, the function should be represented in terms of a graph in the Cartesian coordinate system. In this representation, the function is described and understood as the trace of the fictive non-broken (continuous) movement of an imaginary point in the Cartesian coordinate system. The function is continuous at *c* if the trace of this fictive motion passes *c* and covers the entire area of a neighborhood around *c*. This embodied representation and conceptualization of continuity is heavily reliant on a mental simulation of a fictive motion. The visual system is the key perceptual recourse that is actively employed to ground the concept of continuity into this embodied visual representation. Even in the absence of graphical representation of the function and when the individual thinks about continuity in terms of a fictive motion, the visual system can be employed to re-activate the embodied representation in the mind. This is supported by the findings of studies that have provided evidence suggesting that mental imagery and real visual perception involve the activation of the same areas in the visual system (e.g., Farah, 1989; Bartolomeo, 2008; Hamamé et al., 2012; Liu et al., 2022; Spagna, 2022). Furthermore, since motion is a key element in the embodied representation of continuity, the motor system can also be employed to ground this concept into its embodied representation.

After this brief review of embodied representations of several mathematical concepts, we discuss the embodied representation of infinity. Although the embodiment of infinity shares some similarities with the embodiments of discussed concepts, it has some specific features that need to be discussed in detail.

## 3 Embodiment of infinity

Infinity is one of the key concepts in calculus. Infinity means being unlimited, endless, and boundless (e.g., Ernest, 2023). The word *infinity* may be used in a variety of senses (e.g., Mărăşoiu, 2018). It may refer to the quantity of values. In this sense, any large value can be smaller than a larger value. For example, the question “what is the largest natural number?” does not have a definite answer because any natural number such as *n* is smaller than *n* + *1*. This can be repeated endlessly. In another sense, infinity may refer to the number of elements in sets. For example, the set of natural numbers includes an infinite number of elements. In geometry, infinity may be used in other senses. For example, any line segment can be divided into two smaller line segments, and this process of segmentation can go on infinitely. In another sense of infinity, in projective geometry, two parallel lines can meet one another at infinity. Any sense of infinity may be embodied in a certain way. In this paper, we specifically focus on the embodiment of infinite quantities. Infinite quantities can be divided into infinitely large quantities and infinitely small ones. Infinitely large quantities and infinitely small quantities are not fixed quantities; they are dynamic. While finite real numbers can be represented and embodied in terms of fixed points on an axis of numbers, infinite quantities cannot be represented in terms of fixed points because they cannot be limited by a fixed boundary on the axis. Therefore, embodiment of infinite quantities should be different from the embodiment of finite quantities. A question that is raised here is that how infinite quantities are represented and embodied. Lakoff and Núñez (2000) argue that BASIC METAPHOR OF INFNITY helps us ground our understanding of infinity. In this metaphor, the source domain is a completed iterative process and the target domain is an iterative process that goes on endlessly (Winter and Yoshimi, 2020). Each completed stage of this repeating process has an end point. After the ending of each stage, it is repeated again, and this repetition continues endlessly.

In mathematics discourse, infinitely large quantities are described in terms of unlimited rightward or leftward movements on the axis of numbers. Unlimited movement means that this movement can go beyond any fixed point on the axis. Infinitely large positive quantities (+∞) are described in terms of an unlimited rightward movement that can pass any point on the right side (positive side) of the axis. On the other hand, infinitely large negative quantities (−∞) are described in terms of an unlimited leftward movement that can pass any point on the left side (negative side) of the axis. The key point about the embodiment of this concept is that the movement can pass any fixed point or limit on the axis. Therefore, +∞ is embodied as the rightward movement of an object that goes beyond repeated boundaries that are set on the right side of axis of numbers. Once the moving object has gone beyond one limit, another limit is set on the right side of the previous limit. Then, the moving object passes the latter limit too. This process is repeated again and again.

The embodied conceptualization of +∞ can be realized in gestures. The right hand can be used to represent limits on the right side, and the left hand can be used to represent a moving object that passes the limits. In contrast to the embodiment of many other mathematical concepts, the embodiment of infinity is an endless dynamic process. In embodying +∞, an object moves rightward toward a previously-set limit and passes it. Then, a new limit is set on the right side of the moving object. The moving object continues its movement and passes it as well. This happens again and again. The moving object can pass any limit set on its right side. In other words, there is no impassable limit for it. We call this embodiment as *iterative embodiment*. It is iterative because the process of setting a new limit and passing it is repeated endlessly. This dynamic embodiment takes place over a long period of time consisting of repeated periods, while the embodiment of many mathematical concepts such as arithmetic operations takes place over a much shorter span of time. In the embodiment of infinity large negative quantities, the movement of the object is leftward and the limits are set on the left side of the moving object.

In the process of embodying infinitely large quantities, the visual system and the motor system play important roles, as this process involves spatial concepts and movement. Therefore, it can be suggested that the embodiment of infinitely large quantities takes place through a coordination between visual and motor systems. This iterative embodiment happens over repeated periods of time and can be represented by a set of coordinated gestures that are repeated periodically. Over each period, a limit is set, and then the moving object passes it. The role of gesture in acquiring a grounded understanding of abstract mathematical concepts such as numbers, arithmetic operations, equation of straight line, and many other concepts has been widely discussed in the literature of mathematics education (e.g., Alibali and Nathan, 2012; Khatin-Zadeh et al., 2022a). Since infinitely large quantities can also be embodied in terms of gestural representations, it can be said that the process of acquiring a grounded understanding of infinity can be supported by gestures.

Embodiment of infinitely small quantities has a basic similarity to the embodiment of infinitely large quantities, although it is different in some respects. Infinitely small quantities are dynamic quantities that can become smaller than any tiny quantity. When *x* approaches zero (*x* → 0), the quantity of *x* is infinitely small. When *x* approaches the fixed value *a* (*x* → *a*), the absolute value of *x* subtracted by *a* ( $\left|x-a\right|$ ) is an infinitely small number. This is shown as follows:


$$0<\left|x-a\right|<\varepsilon$$

**Figure 1:**
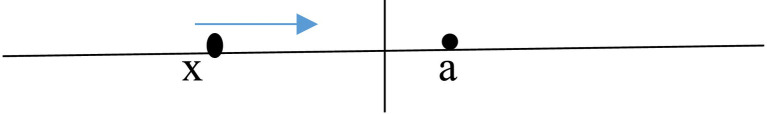
*x* approaches *a* and passes any boundary close to a, but it does not reach *a*.

This means that the difference between *x* and *a* can become infinitely small. In other words, it can become smaller than any tiny value. A question that is raised here is that how this is embodied. Like the embodiment of infinitely large numbers, this is also embodied as a movement. On an axis of numbers, *a* is a fixed point and *x* approaches it from the left side (with values smaller than *a*) or from the right side (with values larger than *a*). In this description, the distance between *x* and *a* can become infinitely small. When *x* approaches *a* from the left side, a boundary is set between *x* and *a*. This boundary is on the right side of *x* and left side of *a* (Figure 1).

The variable of *x* approaches *a* and passes the boundary and continues its movement toward *a*. Then, another boundary is set between *x* and *a*. This new boundary is closer to *a* than the previously-set boundary. The variable of *x* continues its movement toward *a* and passes this new boundary. This iterative process is periodically repeated. The variable of *x* can pass any boundary between *x* and *a*, as the distance between *x* and *a* can become infinitely small. Like the embodiment of infinitely large quantities, the embodiment of infinitely small quantities involves the active role of visual and motor systems, as this embodied description is based on spatial concepts and motion. Therefore, it can be suggested that the embodiment of infinity (infinitely large or small quantities) has a dynamic and iterative nature. It is repeated periodically and endlessly by the active involvement of visual and motor systems.

## 4 Conclusion

Infinity is embodied as a dynamic process that is repeated periodically in an iterative system. This iterative system includes a fixed point (*a*), a moving point (*x*), a dynamic border, and a motion (movement of *x*). All these elements can be represented in terms of a set of coordinated gestures. Therefore, gestures can be actively employed to help students acquire a grounded understanding of infinity and other concepts that are associated with it. This again emphasizes the importance of using gestures in helping students acquire a grounded understanding of mathematical concepts. Some fundamental concepts in calculus such as limit, derivative, integral, tangent line, and series are defined on the basis of infinity. Integral and some infinite series are defined as the sum of infinitely small quantities. In such cases, infinity is used in two senses: infinity as a quantity of a value and infinity of sets. These are perhaps more complex cases that involve more complex embodiment processes. The embodiment of the two senses of infinity may involve different processes. Therefore, when these two senses of infinity are used in a mathematics problem such as an integral problem, two different embodiment processes of infinity can be in operation at the same time. Describing such cases of embodiment of infinity can be the subject of future works.

## Data availability statement

The original contributions presented in the study are included in the article/supplementary material, further inquiries can be directed to the corresponding author.

## Author contributions

OK-Z: Conceptualization, Investigation, Writing – original draft, Writing – review & editing, Formal analysis. DF: Conceptualization, Investigation, Writing – original draft, Writing – review & editing, Funding acquisition. ZE: Writing – review & editing.
